# Health effects of unemployment in Europe (2008–2011): a longitudinal analysis of income and financial strain as mediating factors

**DOI:** 10.1186/s12939-016-0360-6

**Published:** 2016-05-06

**Authors:** Anne Grete Tøge

**Affiliations:** Department of Social Work, Child Welfare and Social Policy, Faculty of Social Sciences, Oslo and Akershus University College of Applied Sciences, P.O. Box 4, St. Olavs plass, NO-0130 Oslo, Norway

**Keywords:** Unemployment, Self-rated health, Income, Financial strain, Europe, Recession, Fixed effects

## Abstract

**Background:**

Unemployment has a number of negative consequences, such as decreased income and poor self-rated health. However, the relationships between unemployment, income, and health are not fully understood. Longitudinal studies have investigated the health effect of unemployment and income separately, but the mediating role of income remains to be scrutinized. Using longitudinal data and methods, this paper investigates whether the effect of unemployment on self-rated health (SRH) is mediated by income, financial strain and unemployment benefits.

**Methods:**

The analyses use data from the longitudinal panel of European Union Statistics on Income and Living Conditions (EU-SILC) over the 4 years of 2008 to 2011. Individual fixed effects models are applied, estimating the longitudinal change in SRH as people move from employment to unemployment, and investigating whether this change is reduced after controlling for possible mediating mechanisms, absolute income change, relative income change, relative income rank, income deprivation, financial strain, and unemployment benefits.

**Results:**

Becoming unemployed is associated with decreased SRH (−0.048, SE 0.012). This decrease is 19 % weaker (−0.039, SE 0.010) after controlling for change in financial strain. Absolute and relative changes in household equalized income, as well as changes in relative rank and transitions into income deprivation, are not found to be associated with change in SRH.

**Conclusions:**

Financial strain is found to be a potential mediator of the individual health effect of unemployment, while neither absolute income, relative income, relative rank, income deprivation nor unemployment benefits are found to be mediators of this relationship.

## Background

The number of unemployed in Europe has increased by more than 10 million since 2008 [1]. As unemployment reduces income for individuals and households [2, 3], and income is assumed to influence the subjective experience of unemployment [4], income changes caused by unemployment could in turn affect health [5, 6]. Reduced income could therefore be an underlying cause of deteriorating health when people become unemployed. The aim of this paper is to contribute to the field by investigating whether and how income and financial strain mediated the health effects of unemployment during the 2008 recession in Europe.

### Mechanisms

The idea that income mediates the health effects of unemployment relies on two assumptions: (1) that unemployment is accompanied by income reduction, and (2) that income reduction has negative health effects. Becoming unemployed entails a shift out of employment and a consequent loss of labour income. In Europe, welfare systems function as substantial buffers against the negative effect of unemployment on income [7, 8]. Nevertheless, the design and regulation of unemployment compensation systems result in some people being defined as eligible whilst others are not. Further, the net replacement rate varies between 13 % (in the United Kingdom) and 92 % (in Portugal), and the duration of the compensation varies between 20 weeks (in Lithuania) and 120 weeks (in Belgium) [7]. Whether unemployment affects income therefore depends on variations in both individual eligibility and national policies. These variations enable investigation of possible mediating health effects of reduced income.

More detrimental health effects of unemployment in people with greater income loss may be explained by different mechanisms [9, 10]. The *absolute income* hypothesis implies that income has a direct effect on health through reduced purchasing power [9, 10] for health-promoting items, services, and activities. However, it is often assumed that this relationship is non-linear: the more health-promoting items, services, and activities that are affordable for the individual or household, the less the additional benefit of affording more [10]. The *income deprivation* hypothesis is a variant of the absolute income hypothesis, but emphasizes the effect of moving below a critical income threshold. Income loss is expected to be harmful if, and only if, it leads to poverty. By acknowledging the role of social comparisons with significant others, the *relative income* hypothesis incorporates the psychosocial dimension of income inequality. Positions in a hierarchical society are seen in relation to both power and social status; being low on the chain of the income distribution can produce feelings of subordination, subservience, and being dominated, which can lead to stress, loss of control, and feelings of inferiority [9–16]. *Relative rank* is closely related to the concept of *relative income*, but implies a stronger emphasis on the psychosocial mechanism than does the relative income hypothesis [9, 10]. Here, health is assumed to be affected not only by the person’s social position relative to a reference group, but also by their position on the scale of income distribution.

### Empirical studies

A wide range of publications report correlations between health and unemployment and/or income. However, investigating whether income mediates the effect of unemployment on health requires investigation of health change within individuals. The research presented in this section is therefore restricted to studies with longitudinal designs.

Several studies suggest a causal relationship between unemployment and health, particularly mental health [19–22], but the effects vary between European countries (see [23] for country specific estimates). Similarly, income is usually found to be strongly correlated with health status [9]. The causal interpretations of such correlations could be that income affects health [24, 25], that health affects income [25], that income and health affect each other mutually, or that underlying factors cause both [26, 27]. Interpretations may also depend on the definition of “causal relationship” as well as the investigated sample [9]. For example, income may have a negative effect on health among children and most adults, but not in those over the age of 70 years [28].

Using structural equation models, income deprivation at the household level is found to predict forthcoming health problems [25]. Testing the inverse causal pathway shows the effects of health on household resources to be markedly lower than the other way around, supporting the hypothesis that income is an important determinant of health. Halleröd and Gustafsson [29] use similar models and report that changes in income are also related to changes in morbidity, but they are not able to determine the causal direction. Investigating how more severe income shocks affect health using dynamic panel models, Halliday [30] reports that income shock is on average associated with deteriorating SRH. Conversely, changes towards the middle of the income distribution are associated with increased SRH for those with both very high and very low income.

Very few studies have investigated how income and financial strain mediate the health effect of unemployment, but in a recent study Huijts, Reeves, McKee and Stuckler [31] address this exact question using the EU-SILC (2007–2009) and conclude that self-reported financial strain could explain about one third of the association between job loss and health. However, Huijts*,* et al*.* [31] do not investigate changes over time, but use a control for the baseline. These models are prone to omitted-variable bias due to baseline differences in working conditions, stress, or job insecurity, which are likely to affect the risk of unemployment, income change, and health. Such bias leads to overestimation of the health effects of unemployment and income change, and increases the risk of overestimating the mediating effect. This illustrates the need for longitudinal investigation.

Further, there are many reasons for income fluctuations, e.g. more/less working hours or getting a better/worse paid job. Such income changes should neither cause better nor worse health. When investigating how income mediates the effect of unemployment on health, one should therefore investigate the patterns among individuals who have experienced unemployment rather than the correlations in the general population.

## Methods

### Data

This analysis uses the 2008–2011 panel of European Union Statistics on Income and Living Conditions (EU-SILC), which covers 28 European countries (the EU-28 except for Germany and Ireland plus Norway and Iceland). The data are harmonized according to the European Parliament and Council regulation (1177/2003) and constitute an extraordinarily rich information source on employment.

### Variables

The dependent variable is SRH, measured with the single item: How is your health in general? The responses are captured on a 5-point scale ranging from 1 (very bad) to 5 (very good). This measure is more sensitive to minor health changes than longstanding illness or chronic disease. A continuous measure of health provides more variation than a dichotomised measure of health, and linear regression models allow for more straightforward comparisons between countries and statistical models than non-linear regression of categorical outcome variables. Empirical research finds SRH a powerful predictor of future morbidity, mortality [32–34], and future health ratings from physicians [35, 36].

Unemployment is given the value 1 if a respondent’s self-defined status is unemployed, and 0 if it is employed. All other statuses are coded as missing.

*Absolute income* is measured as log (income + 1), where income is the net sum (in thousands of €) of disposable household income, including welfare benefits and minus fixed costs (housing, utilities, debts, etc.), and adjusted for inflation (Harmonised Index of Consumer Prices [37]) and household size (OECD equivalence scale [38]). The equivalence scale assigns a value of 1 to the respondent, 0.5 to each additional adult member. and 0.3 to each child [39, 40]. *Income deprivation* is measured as a key measure of poverty in the EU list of indicators [41]. The “at-risk-of-poverty” threshold is set at 60 % of national median household income [42]. Living below this cut-off is coded 1 and above is coded 0. *Relative income* is measured as deviation between household absolute income and country/year median [10]. Income changes are therefore adjusted for changes in the overall income level in the national population. *Relative rank* is measured as the households’ position within the national distribution of household absolute incomes [10]. This distribution is separated into deciles, where 1 denotes the 20 %with lowest income and 5 denotes the 20 % with highest income. The subjective dimension of the households’ *economic difficulties* is measured on a 6-item scale of their ability to meet their needs, where 1 is very easy and 6 very difficult.

To investigate the independent mediating effect of unemployment benefits on SRH, net unemployment benefit is extracted from the absolute income and log (net unemployment benefit + 1) and log (absolute income − net unemployment benefit + 1) are included as independent variables. Gross unemployment benefit is used for countries where net unemployment benefits are unavailable (Denmark, Finland, Hungary, Iceland, Malta, Netherlands, Norway, Slovakia, and the United Kingdom).

#### Control variables

Age is controlled for using linear (years) and squared terms. Partnership status is controlled for using an indicator variable for married and cohabiting individuals (1) versus all other statuses (0).

### Sample restrictions

EU-SILC is a 4-year rotational panel of national representative samples. However, in this study only people aged 19 to 65 with at least 2 years in the labour market (employed and/or unemployed) and at least one transition to unemployment are included. People from Croatia were observed only once (because Croatia joined EU-SILC in 2011) and are therefore excluded from the analysis. To avoid introducing reverse causality (i.e. the effect of health on income and unemployment), the sample is restricted to those with less than 3 months of absence or disability in the year prior to the transition to unemployment. Because household income depends on all household members, people who moved households in this period are also excluded from the analyses. The final sample includes 16 913 individual observations among 6 200 respondents.

### Statistical analyses

Individual fixed effects models, i.e. models that control for time-invariant factors, are applied. This is a form of difference-in-difference design with a model that contrasts the health slope for those who experience unemployment with those who do not. Random models are not applicable, as the Hausman test showed statistical dependence between explanatory variables and the unobserved random term. In the fixed effects model individual change in SRH is a function of change in the explanatory variables. The basic model is

$$ {Y}_{\mathrm{i}\mathrm{t}}={\upmu}_{\mathrm{t}}+\upbeta \kern0.2em {x}_{\mathrm{i}\mathrm{t}}+{\upnu}_{\mathrm{i}}+{\upvarepsilon}_{\mathrm{i}\mathrm{t}\kern.35em }\mathrm{f}\mathrm{o}\mathrm{r}\kern0.5em \mathrm{t}=1,\dots, \mathrm{T}\kern0.5em \mathrm{and}\kern0.5em \mathrm{i}=1,\dots, \mathrm{N}(0) $$

where *y*_it_ is the value of SRH for unit *i* at time *t*, μ_t_ is an intercept that may be different for each period, and β*x*_it_ is the value of the explanatory variable(s) for unit *i* at time *t*. As the models only use the within-individual variation, they control for unobserved factors that vary across units but are constant over time; ν_i_. ε_it_ is the unobserved time variant factor (error term).

All the main mediating variables are included separately, since different measures of income are highly correlated [10]. Combining them in one model would introduce multicolinearity [43].

Partnership status and number of dependent children are controlled for by equalizing disposable household income. To avoid multicollinearity, control for partnership status and children are only included in investigations of the impact of financial strain; reemployment is not included because this transition correlates with income change. All standard errors are clustered on countries. The analyses were conducted using Stata/MP 14.

## Results

Table 1 reports summary statistics. Standard deviations are reported for individuals and show variation in individual change over time.

**Table 1:** Summary statistics

Variables	Mean	SD (within)	Min	Max	*N*
Dependent variable:					
Self-rated health (SRH)	3.90	0.43	1	5	16,913
Employment:					
Unemployment	0.41	0.47	0	1	16,913
Equalized disposable household income:					
Absolute income	2.23	0.25	−2.98	5.73	16,913
Income deprivation	>0.01	0.05	0	1	16,913
Relative income	−0.11	0.25	−5.82	3.12	16,913
Relative rank	2.69	0.63	1	5	16,913
Absolute income – excluding unemployment benefit	2.19	0.27	−2.98	5.73	16,913
Net unemployment benefit	0.33	0.44	0	5.12	16,913
Subjective perception of economy:					
Financial strain	4.44	0.60	1	6	16,913
Time variant covariates:					
Partnered	0.60	0.10	0	1	16,913
Age (in years)	39.29	0.87	19	59	16,913

### Income and SRH

In all European countries, transition from employment to unemployment implies lower income [7]. Except for income deprivation, this pattern can be rediscovered for all income and material factors included in this study (Appendix, Table 4). Table 2 reports individual fixed effects correlations, where SRH is a function of income and material factors. Models 1a and 2a show that increased *absolute* and *relative income* is associated with increased SRH. However, neither of these two estimates are significant. Model 3a investigates individual change in SRH as a function of change in *relative rank*, and shows that upward mobility in income distribution is associated with increased SRH, but this correlation is not statistically significant. Model 4a shows that moving into income deprivation (below 60 % of national median household income), is associated with a positive, but statistically insignificant change in SRH. Model 5a shows that increased financial strain is significantly correlated with deterioration in SRH: for each increase in the level of financial strain, SRH score drops by 0.044. By separating benefits from income, Model 6a investigates the effect of unemployment benefits beyond their effect on income. Results show that increased unemployment benefit is associated with a positive, but statistically insignificant increase in SRH.

**Table 2:** Self-rated health (SRH): Individual fixed effects correlations. All models control for age and age squared

	Model 1a	Model 2a	Model 3a	Model 4a	Model 5a	Model 6a
	Fixed effects	Fixed effects	Fixed effects	Fixed effects	Fixed effects	Fixed effects
Variables	SRH	SRH	SRH	SRH	SRH	SRH
Absolute income	0.006					
	(0.013					
Relative income		0.009				
		(0.013)				
Relative rank			0.003			
			(0.004)			
Income deprivation				0.010		
				(0.106)		
Financial strain					−0.044***	
					(0.009)	
Absolute income – excluding unemployment benefit						0.005
						(0.014)
Unemployment benefit						0.013
						(0.011)
Control for partnership status:						
	NO	NO	NO	NO	YES	NO
Observations	16,913	16,913	16,913	16,913	16,913	16,912
R-squared (within)	0.009	0.009	0.009	0.009	0.013	0.009
Number of respondents	6,200	6,200	6,200	6,200	6,200	6,200

Standard errors clustered on countries in parentheses, ***p < 0.001, **p < 0.01, *p < 0.05

### Income mediation

The results in Table 2 show that only financial strain (Model 5a) affects SRH, implying that only financial strain can be expected to be a significant mediating effect between unemployment and SRH. Nevertheless, for transparency, Table 3 reports results for all models.

**Table 3:** Self-rated health (SRH): individual fixed effects correlations. All models control for age and age squared

	Model 0	Model 1b	Model 2b	Model 3b	Model 4b	Model 5b	Model 6b
	Fixed effects	Fixed effects	Fixed effects	Fixed effects	Fixed effects	Fixed effects	Fixed effects
Variables	SRH	SRH	SRH	SRH	SRH	SRH	SRH
Unemployment	−0.048***	−0.048***	−0.048***	−0.048***	−0.048***	−0.039***	−0.050***
	(0.010)	(0.010)	(0.010)	(0.010)	(0.010)	(0.010)	(0.009)
Absolute income		0.001					
		(0.014)					
Relative income			0.005				
			(0.014)				
Relative rank				0.002			
				(0.004)			
Income deprivation					0.016		
					(0.109)		
Financial strain						−0.041***	
						(0.009)	
Absolute income – excluding unemployment benefit							>0.001
							(0.014)
Unemployment benefit							0.017
							(0.011)
Control for partnership status:							
	YES	NO	NO	NO	NO	YES	NO
Observations	16,913	16,913	16,913	16,913	16,913	16,913	16,912
R-squared (within)	0.011	0.010	0.010	0.010	0.010	0.014	0.010
Number of respondents	6,200	6,200	6,200	6,200	6,200	6,200	6,200

Standard errors clustered on countries in parentheses, *** *p* < 0.001, ** *p* < 0.01, * *p* < 0.05

Model 0 reports a mean reduction in SRH of 0.048 when respondents become unemployed. Change in absolute income (Model 1b), relative income (Model 2b), relative rank (Model 3b), and income deprivation (Model 4b) does not substantially affect the unemployment coefficient; the mean reduction in SRH when respondents become unemployed is 0.047 (in models 1b, 2b and 3b) and 0.048 (in Model 4b). However, when controlling for financial strain (Model 5b), the unemployment coefficient is −0.039, in other words 19 % lower than the unemployment coefficient in Model 0. Nevertheless, a bootstrap estimation (50 replications) does not suggest that the unemployment estimate in Model 5b is significantly different from that in Model 0 (CI = −.019-037).

Model 6b investigates the mediating effect of unemployment benefits, but shows a minor *increase* in the unemployment coefficient, and can therefore not identify a mediating effect.

The results on Table 3 show a possible mediating effect of financial strain (Model 5b), however, it cannot be concluded that this mediating effect is different from zero. No mediating effects are detected from the remaining dimensions of income.

### Sensitivity analyses

To test whether the results in Models 6a and 6b are robust to the inclusion of gross unemployment benefits, they are rerun on a sample restricted to individuals in countries where net unemployment benefits are available (see Appendix, Table 5). The main result persists: the mean change in SRH when respondents become unemployed does not decline when controlling for net unemployment benefit. If anything, there is rather a stronger effect of unemployment on SRH.

Tøge & Blekesaune [23] found stronger effects of unemployment on SRH among older than younger workers. When limiting the analyses in the current study to individuals born before 1970, results confirm the main finding. Only financial strain reduces the unemployment estimate (Appendix, Table 6), however, bootstrap estimation suggests that the reduction is not statistically significant (CI = −.030–.055).

**Fig. 1:**
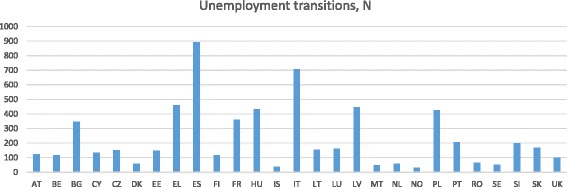
Number of respondents with unemployment transitions, N per country

The number of respondents with unemployment transitions varies substantially across countries (see Fig. 1). Using fixed effects models, this variation implies that the results could be driven by effects in countries with high numbers of unemployment transitions.

Weighting for these differences, either by giving the countries even numbers of transitions (i.e. $\frac{1}{\mathrm{Number}\ \mathrm{of}\ \mathrm{unemployment}\ \mathrm{transitions}}$, see Appendix, Table 7) or by weighting according to the national increase in unemployment rates (i.e. $\frac{\Delta\ \mathrm{N}\mathrm{o}\mathrm{n}\ \mathrm{employment}\ \mathrm{rate}\ \left(2008\hbox{--} 2011\right)}{\mathrm{Number}\ \mathrm{o}\mathrm{f}\ \mathrm{unemployment}\ \mathrm{transitions}}$, see Appendix, Table 8) confirms the overall results. Differences between Model 0 and 5b in Appendix Tables 6 and 7 are not tested, as weights are not allowed with the bootstrap prefix in Stata/MP 14.

## Discussion

This study provides a longitudinal investigation of income and financial strain as mediators of the health effect of unemployment in the first years after the global financial crisis hit Europe. The main results suggest that the effect of unemployment on SRH is more or less independent of change in income, but possibly somewhat mediated by self-perceived financial strain.

Huijts*,* et al*.* [31] investigated the potential mediating role of income and financial strain in the EU-SILC using a cross-sectional model that controls for initial health. This method provides estimates between cross-sectional and longitudinal estimates. In this case, a cross-sectional estimate is several times larger than a longitudinal estimate [23], indicating that the health gap between working and unemployed individuals is largely driven by health selection of individuals in poor health into unemployment and much less by changing health as people become unemployed. Longitudinal models that investigate changes in health in individuals remove all time-invariant sources of health selection into unemployment, and thus account for the fact that people in poor health are more likely to become unemployed than healthier individuals.

The economic consequences of unemployment are influenced by the functioning of the welfare state, including the level and duration of benefits and the access to services during unemployment [4]. Despite variation across countries, European welfare states have performed considerably better than the United States (US) during this period [44]. Because income reductions in Europe are typically better ameliorated by benefits and services than in the US, the negative consequences of individual and family income loss in Europe may be lower. This could mean that different forms of compensation, insurance, and benefits that have been provided throughout Europe have been quite effective in buffering the health effects of unemployment in this critical period. However, it is important to note that this study is based on a 4-year observational period. Even though psychological stress could be immediate [45] or even start before the job loss [46], the effects of actual income loss on the social determinants of health may take more than a few years to manifest [4]. Duration of unemployment and period of lower income may therefore be more important than actual income change at the time of transition to unemployment. Further, the panel does not include the years after 2011, when several countries introduced austerity measures. To the extent that such measures include cutbacks in benefits and services for the unemployed, income could become a stronger mediating factor.

The effect of unemployment on health could also depend on the time interval between unemployment transition and interview. When empirically tested, SRH tends to rise after the unemployment transition [23], indicating a gradually health improvement or adaptation to the new situation. However, the timing of interview should be independent of the timing of unemployment. As such, the coefficients indicate the change in SRH for a person with an average time interval between unemployment and interview.

Beyond including absolute income as a mediating factor, this study attempted to test other mechanisms by which income might mediate the health effect of unemployment: relative income, relative rank, and income deprivation. The results provide no evidence for any of these pathways. Disentangling the different income-related mechanisms is difficult, as the chain of events is complex [9, 47] and the operationalization of the various income measures does not necessary exclude alternative hypotheses [10]. Economic resources affect living conditions in absolute terms, but the extent to which material factors directly affect health is difficult to separate from indirect pathways through health behaviours, low control, insecurity, loss of self-esteem, and social isolation [18, 47]. “Usually the effects of chronic stress will be closely related to the many direct effects of material deprivation, simply because material insecurity is always worrying” [18]. Such psychological effects could be related to the various systems of compensation, insurance and benefits for the unemployed. Beyond buffering income reductions, unemployment protection might also contribute to a perception of safety and increase trust in the state as a provider of welfare and social security. Given that such emotions affect health, there could be an independent psychological effect of compensation on health and wellbeing; however, this study finds no evidence for this mechanism as a mediating effect of unemployment on health.

Subjective assessments of one’s financial situation may shed light on another psychosocial pathway: self-perceived economic stress [11, 17], a dimension that is not necessarily captured by objective measures of income change [21]. When measuring peoples’ abilities to subsist on their current income, it is necessary to have their subjective judgement of their present financial situation. This judgement, however, relates to their perceived future economic prospects [48]. Even in a household with a stable income and ability to maintain its normal standard of living, risk of upcoming change in income may affect a person’s consumption and judgement of their current financial situation. In line with Huijts*,* et al*.* [31], the present results indicate that financial strain could mediate the effect of unemployment on health; the estimated size of this mediating effect is 19 %, i.e. about half of what Huijts*,* et al*.* [31] suggest. Nevertheless, the mediating effect of financial strain is not statistically significantly different from zero. It should also be mentioned that these 19 % are estimated without considering possible endogeneity: with self-reported measurements on both sides of the equation, there is the risk that time-variant psychological processes may influence both the dependent and independent variables. An underlying variable, e.g. mood, could affect both subjective economy and SRH. This means that the “true” mediating effect of financial strain in the European population during the financial crisis (2008–2011) would be somewhere below 19 %.

Contrary to Huijts*,* et al*.* [31], this study finds no mediating effect of unemployment benefits. This result does not necessarily mean that health is unaffected by such schemes; it could rather illustrate the difficulty of identifying such effects. By grouping all income sources into one pot, it is possible to examine the health effects of income changes. This pooling of income relies on the assumption that unemployment with low (or no) compensation would give a steeper decline in overall household income than unemployment with compensation. However, lack of compensation for one household member could be an incentive for higher work intensity among other others, and consequently generate a higher overall household income. When one person becomes unemployed, other household members can be a substantial buffer against income reductions. Isolating the effect of unemployment benefits may therefore be difficult; increased unemployment benefits at the household level could simply imply that more members are unemployed.

### Strengths and limitations

All analyses are longitudinal estimates drawn from the EU-SILC panel, which includes data on income and living conditions for almost 17,000 Europeans who experienced a transition to unemployment in the years 2008 to 2011. If the health effects of unemployment are mediated by income, evidence should be findable in these data.

It is important to note that SRH is a crude measurement of health. Unemployment transition could have diverging effects on mental and physical health [49–52]. Although SRH might be more sensitive to mental health than more specific measures of illness or health conditions, it is not possible to separate these effects. More fine-grained health measures are preferable, but unfortunately not available in the EU-SILC.

Whether income mediates the effect of unemployment on health could depend on the position within the labour market. However, such analyses are not possible due to limited information in the EU-SILC.

Attrition is a problem in longitudinal survey data and could affect the results. The rotational design of EU-SILC does not provide necessary information to address the impact of attrition biases. Emigrating respondents are followed until they emigrate, but not after. If emigration is more prevalent among people who experience stronger (or weaker) health effects of reduced income following unemployment, emigration will bias the estimates.

## Conclusion

Changes in both absolute and relative income, as well as in self-reported financial strain, are significantly related to changes in SRH; however, only financial strain is found to be a potential mediator of the individual health effect of unemployment.

## Appendix

**Table 4:** Income and material factors as functions of unemployment. All models control for age and age squared

	Model A	Model B	Model C	Model D	Model E	Model F	Model G
	Fixed effects	Fixed effects	Fixed effects	Fixed effects	Fixed effects	Fixed effects	Fixed effects
Variables	Absolute income	Relative income	Relative rank	Income deprivation	Financial strain	Absolute income – excl. unemp. benefit	Unemployment benefit
Unemployment	−0.055*	−0.040*	−0.061*	−0.002	−0.217***	−0.070***	0.138***
	(0.013)	(0.013)	(0.027)	(0.001)	(0.020)	(0.015)	(0.029)
Control for partnership status:	NO	NO	NO	NO	NO	YES	NO
Observations	16,913	16,913	16,913	16,913	16,913	16,913	16,912
R-squared (within)	0.016	0.018	0.015	0.015	0.002	0.049	0.108
Number of respondents	6,200	6,200	6,200	6,200	6,200	6,200	6,200

Standard errors clustered on countries in parentheses, *** *p* < 0.001, ** *p* < 0.01, * *p* < 0.05

**Table 5:** Sensitivity test, restricted to individuals in countries where net unemployment benefit is available. All models control for age and age squared

	Model 0	Model 6a	Model 6b
	Fixed effects	Fixed effects	Fixed effects
Variables	SRH	SRH	SRH
Unemployment	−0.056***		−0.059***
	(0.011)		(0.010)
Absolute income – excluding unemployment benefit		−0.006	−0.011
		(0.011)	(0.012)
Unemployment benefit		0.014	0.018
		(0.012)	(0.02)
Control for partnership status:			
	YES	NO	NO
Observations	14,083	14,083	14,083
R-squared (within)	0.009	0.007	0.009
Number of respondents	5,151	5,151	5,151

Standard errors clustered on countries in parentheses, *** *p* < 0.001, ** *p* < 0.01, * *p* < 0.05

**Table 6:** Sensitivity test. Restricted to respondents born before 1970

	Model 0	Model 1b	Model 2b	Model 3b	Model 4b	Model 5b	Model 6b
	Fixed effects	Fixed effects	Fixed effects	Fixed effects	Fixed effects	Fixed effects	Fixed effects
Variables	SRH	SRH	SRH	SRH	SRH	SRH	SRH
Unemployment	−0.074*	−0.073*	−0.074*	−0.074*	−0.074*	−0.061*	−0.075*
	(0.014)	(0.014)	(0.014)	(0.014)	(0.014)	(0.015)	(0.015)
Absolute income		0.004					
		(0.023)					
Relative income			−0.004				
			(0.025)				
Relative rank				0.004			
				(0.008)			
Income deprivation					0.113		
					(0.162)		
Financial strain						−0.053***	
						(0.009)	
Absolute income – excluding unemployment benefit							0.003
							(0.021)
Unemployment benefit							0.005
							(0.013)
Control for partnership status:							
	YES	NO	NO	NO	NO	YES	NO
Observations	8,249	8,249	8,249	8,249	8,249	8,249	8,249
R-squared (within)	0.016	0.016	0.016	0.017	0.017	0.022	0.016
Number of respondents	2,966	2,966	2,966	2,966	2,966	2,966	2,966

Standard errors clustered on countries in parentheses, *** *p* < 0.001, ** *p* < 0.01, * *p* < 0.05

**Table 7:** Sensitivity test. Weighted for uneven numbers of unemployment transitions. All models control for age and age squared

	Model 0	Model 1b	Model 2b	Model 3b	Model 4b	Model 5b	Model 6b
	Fixed effects	Fixed effects	Fixed effects	Fixed effects	Fixed effects	Fixed effects	Fixed effects
Variables	SRH	SRH	SRH	SRH	SRH	SRH	SRH
Unemployment	−0.044**	−0.042	−0.043	−0.043**	−0.044**	−0.027	−0.044*
	(0.021)	(0.021)	(0.021)	(0.021)	(0.021)	(0.020)	(0.021)
		0.020					
		(0.035)					
Relative income			0.017				
			(0.033)				
Relative rank				0.003			
				(0.007)			
Income deprivation					0.131		
					(0.166)		
Financial strain						−0.059***	
						(0.014)	
Absolute income – excl. unemployment benefit							0.015
							(0.034)
Unemployment benefit							0.010
							(0.015)
Control for partnership status:							
	YES	NO	NO	NO	NO	YES	NO
Observations	16,913	16,913	16,913	16,913	16,913	16,913	16,912
R-squared (within)	0.009	0.009	0.009	0.009	0.009	0.013	0.009
Number of respondents	6,200	6,200	6,200	6,200	6,200	6,200	6,200

Standard errors clustered on countries in parentheses, *** *p* < 0.001, ** *p* < 0.01, * *p* < 0.05

**Table 8:** Sensitivity test. Weighted for uneven numbers of Unemployment s and change in unemployment rate (2008–2011). All models control for age and age squared

	Model 0	Model 1b	Model 2b	Model 3b	Model 4b	Model 5b	Model 6b
	Fixed effects	Fixed effects	Fixed effects	Fixed effects	Fixed effects	Fixed effects	Fixed effects
Variables	SRH	SRH	SRH	SRH	SRH	SRH	SRH
Unemployment	−0.041*	−0.040	−0.040	−0.041*	−0.041*	−0.026	−0.042*
	(0.020)	(0.020)	(0.020)	(0.020)	(0.020)	(0.019)	(0.020)
Absolute income		0.025					
		(0.038)					
Relative income			0.022				
			(0.035)				
Relative rank				0.004			
				(0.007)			
Income deprivation					0.126		
					(0.161)		
Financial strain						−0.057***	
						(0.013)	
Absolute income – excluding unemployment benefit							0.021
							(0.037)
Unemployment benefit							0.012
							(0.016)
Control for partnership status:							
	YES	NO	NO	NO	NO	YES	NO
Observations	16,913	16,913	16,913	16,913	16,913	16,913	16,912
R-squared (within)	0.009	0.010	0.010	0.009	0.009	0.017	0.010
Number of respondents	6,200	6,200	6,200	6,200	6,200	6,200	6,200

Standard errors clustered on countries in parentheses, *** *p* < 0.001, ** *p* < 0.01, * *p* < 0.05
